# Supplementary material for: Sequestration of Sulfate Anions from Groundwater by Biopolymer-Metal Composite Materials
Source: Polymers (Basel). 2020 Jul 6;12(7):1502. doi: 10.3390/polym12071502 (PMC7408214; doi:10.3390/polym12071502)
Supplement: Supplementary file 1 [file polymers-12-01502-s001.pdf]

*Supporting Information*

# Sequestration of Sulfate Anions from Groundwater by Biopolymer-Metal Composite Materials

**Md. Mehadi Hassan [1,2,†], Mohamed H. Mohamed [1,†], Inimfon A. Udoetok [1,†], Bernd G. K. Steiger [1] and Lee D. Wilson [1,*]**

[1]Department of Chemistry, University of Saskatchewan, 110 Science Place, Saskatoon, SK S7N 5C9, Canada; mehadibdbaust@gmail.com (M.M.H.); mom133@mail.usask.ca (M.H.M.); inu850@mail.usask.ca (I.A.U.); bes241@usask.ca (B.G.K.S.)

[2]Department of Arts and Sciences, Bangladesh Army University of Science and Technology, Saidpur 5311, Bangladesh;

* Correspondence: lee.wilson@usask.ca; Tel.: +1-306-966-2961; Fax: +1-306-966-4730

† Denotes equal authorship contributions to this work.

*Supporting Information*

**Table S1.** Chemical analysis report of Well-1 sample.

| Analysis | Result | Unit | Sask Guideline |
|---|---|---|---|
| Health and Toxicity Panel<br>Boron | 0.1 | mg/L | < 5.0 |
| Aluminum (ICPMS) | <6.96 | µg/L | No Guideline |
| Arsenic (ICPMS) | 0.60 | µg/L | < 10 |
| Barium (ICPMS) | 18.8 | µg/L | < 1000 |
| Cadmium (ICPMS) | <0.15 | µg/L | < 5 |
| Chromium (ICPMS) | <0.19 | µg/L | < 50 |
| Copper (ICPMS) | <8.29 | µg/L | < 1000 |
| Lead (ICPMS) | 0.10 | µg/L | < 10 |
| Selenium (ICPMS) | 5.8 | µg/L | < 10 |
| Uranium (ICPMS) | 34.4 | µg/L | < 20 |
| Zinc (ICPMS) | 7.2 | µg/L | < 5000 |
| Antimony (ICPMS) | <0.16 | µg/L | No Guideline |
| Silver (ICPMS) | <0.20 | µg/L | No Guideline |
| General Chemistry/Water Quality Panel | | | |
| Conductivity | 3711 | µS/cm | < 2300 |
| pH | 7.7 | pH Units | 6.5 - 9.0 |
| Total Alkalinity | 346 | mg/L CaCO3 | < 500 |
| Phenol Alkalinity | 0.00 | mg/L CaCO3 | No Guideline |
| Bicarbonate | 422 | mg/L | No Guideline |
| Carbonate | 0 | mg/L | No Guideline |
| Hydroxide | 0 | mg/L | No Guideline |
| Chloride Dissolved | 98.3 | mg/L | < 250 |
| Fluoride Dissolved | 0.24 | mg/L | < 1.5 |
| Nitrate Dissolved | 6.9 | mg/L | < 45 |
| Sulfate Dissolved | 2062.6 | mg/L | < 500 |
| Total Hardness (Calculated) | 1912 | mg/L CaCO3 | < 800 |
| Total Dissolved Solids | 3552 | mg/L | < 1500 |
| Iron | <0.1 | mg/L | < 0.3 |
| Manganese | <0.01 | mg/L | < 0.05 |
| Calcium | 388 | mg/L | No Guideline |
| Magnesium | 229 | mg/L | < 200 |
| Potassium | 6 | mg/L | No Guideline |
| Sodium | 339 | mg/L | < 300 |

**Table S2.** Chemical analysis report of Well-2 sample.

| Analysis | Result | Unit | Sask Guideline |
|---|---|---|---|
| Health and Toxicity Panel Boron | 0.3 | mg/L | < 5.0 |
| Aluminum (ICPMS) | 36.6 | µg/L | No Guideline |
| Arsenic (ICPMS) | 0.90 | µg/L | < 10 |
| Barium (ICPMS) | 14.7 | µg/L | < 1000 |
| Cadmium (ICPMS) | <0.15 | µg/L | < 5 |
| Chromium (ICPMS) | 0.30 | µg/L | < 50 |
| Copper (ICPMS) | <8.29 | µg/L | < 1000 |
| Lead (ICPMS) | 0.40 | µg/L | < 10 |
| Selenium (ICPMS) | 3.3 | µg/L | < 10 |
| Uranium (ICPMS) | 94.4 | µg/L | < 20 |
| Zinc (ICPMS) | 18.6 | µg/L | < 5000 |
| Antimony (ICPMS) | <0.16 | µg/L | No Guideline |
| Silver (ICPMS) | <0.20 | µg/L | No Guideline |
| General Chemistry/Water Quality Panel | | | |
| Conductivity | 4978 | µS/cm | < 2300 |
| pH | 7.8 | pH Units | 6.5 - 9.0 |
| Total Alkalinity | 486 | mg/L CaCO3 | < 500 |
| Phenol Alkalinity | 0.00 | mg/L CaCO3 | No Guideline |
| Bicarbonate | 593 | mg/L | No Guideline |
| Carbonate | 0 | mg/L | No Guideline |
| Hydroxide | 0 | mg/L | No Guideline |
| Chloride Dissolved | 146.8 | mg/L | < 250 |
| Fluoride Dissolved | 0.29 | mg/L | < 1.5 |
| Nitrate Dissolved | 27.6 | mg/L | < 45 |
| Sulfate Dissolved | 2653.7 | mg/L | < 500 |
| Total Hardness (Calculated) | 2198 | mg/L CaCO3 | < 800 |
| Total Dissolved Solids | 4711 | mg/L | < 1500 |
| Iron | <0.1 | mg/L | < 0.3 |
| Manganese | <0.01 | mg/L | < 0.05 |
| Calcium | 331 | mg/L | No Guideline |
| Magnesium | 333 | mg/L | < 200 |
| Potassium | 8 | mg/L | No Guideline |
| Sodium | 618 | mg/L | < 300 |

**Table S3.** Chemical analysis report of Well-3 sample.

| Analysis | Result | Unit | Sask Guideline |
|---|---|---|---|
| Health and Toxicity Panel<br>Boron | 0.1 | mg/L | < 5.0 |
| Aluminum (ICPMS) | <6.96 | µg/L | No Guideline |
| Arsenic (ICPMS) | 2.8 | µg/L | < 10 |
| Barium (ICPMS) | 19.2 | µg/L | < 1000 |
| Cadmium (ICPMS) | 0.80 | µg/L | < 5 |
| Chromium (ICPMS) | 0.20 | µg/L | < 50 |
| Copper (ICPMS) | <8.29 | µg/L | < 1000 |
| Lead (ICPMS) | 0.10 | µg/L | < 10 |
| Selenium (ICPMS) | 3.5 | µg/L | < 10 |
| Uranium (ICPMS) | 48.4 | µg/L | < 20 |
| Zinc (ICPMS) | <4.00 | µg/L | < 5000 |
| Antimony (ICPMS) | <0.16 | µg/L | No Guideline |
| Silver (ICPMS) | <0.20 | µg/L | No Guideline |
| General Chemistry/Water Quality Panel | | | |
| Conductivity | 9515 | µS/cm | < 2300 |
| pH | 7.5 | pH Units | 6.5 - 9.0 |
| Total Alkalinity | 592 | mg/L CaCO3 | < 500 |
| Phenol Alkalinity | 0.00 | mg/L CaCO3 | No Guideline |
| Bicarbonate | 722 | mg/L | No Guideline |
| Carbonate | 0 | mg/L | No Guideline |
| Hydroxide | 0 | mg/L | No Guideline |
| Chloride Dissolved | 328.1 | mg/L | < 250 |
| Fluoride Dissolved | 0.13 | mg/L | < 1.5 |
| Nitrate Dissolved | 1.6 | mg/L | < 45 |
| Sulfate Dissolved | 6030.0 | mg/L | < 500 |
| Total Hardness (Calculated) | 4194 | mg/L CaCO3 | < 800 |
| Total Dissolved Solids | 9896 | mg/L | < 1500 |
| Iron | <0.1 | mg/L | < 0.3 |
| Manganese | 3.69 | mg/L | < 0.05 |
| Calcium | 593 | mg/L | No Guideline |
| Magnesium | 659 | mg/L | < 200 |
| Potassium | 49 | mg/L | No Guideline |
| Sodium | 1513 | mg/L | < 300 |

*Supporting Information*

**Table S4.** Sips isotherm adsorption parameters for sulfate dianion species at 298 K and pH 6.8 in aqueous solution.

| Sorbent | $Q_m$ (mg/g) | $K_s$ | $n_s$ | $R^2$ |
|---|---|---|---|---|
| Chitosan | 288.1 | 0.0006 | 1.5831 | 0.98 |
| CACu1 | 371.4 | 0.0012 | 2.4599 | 0.99 |

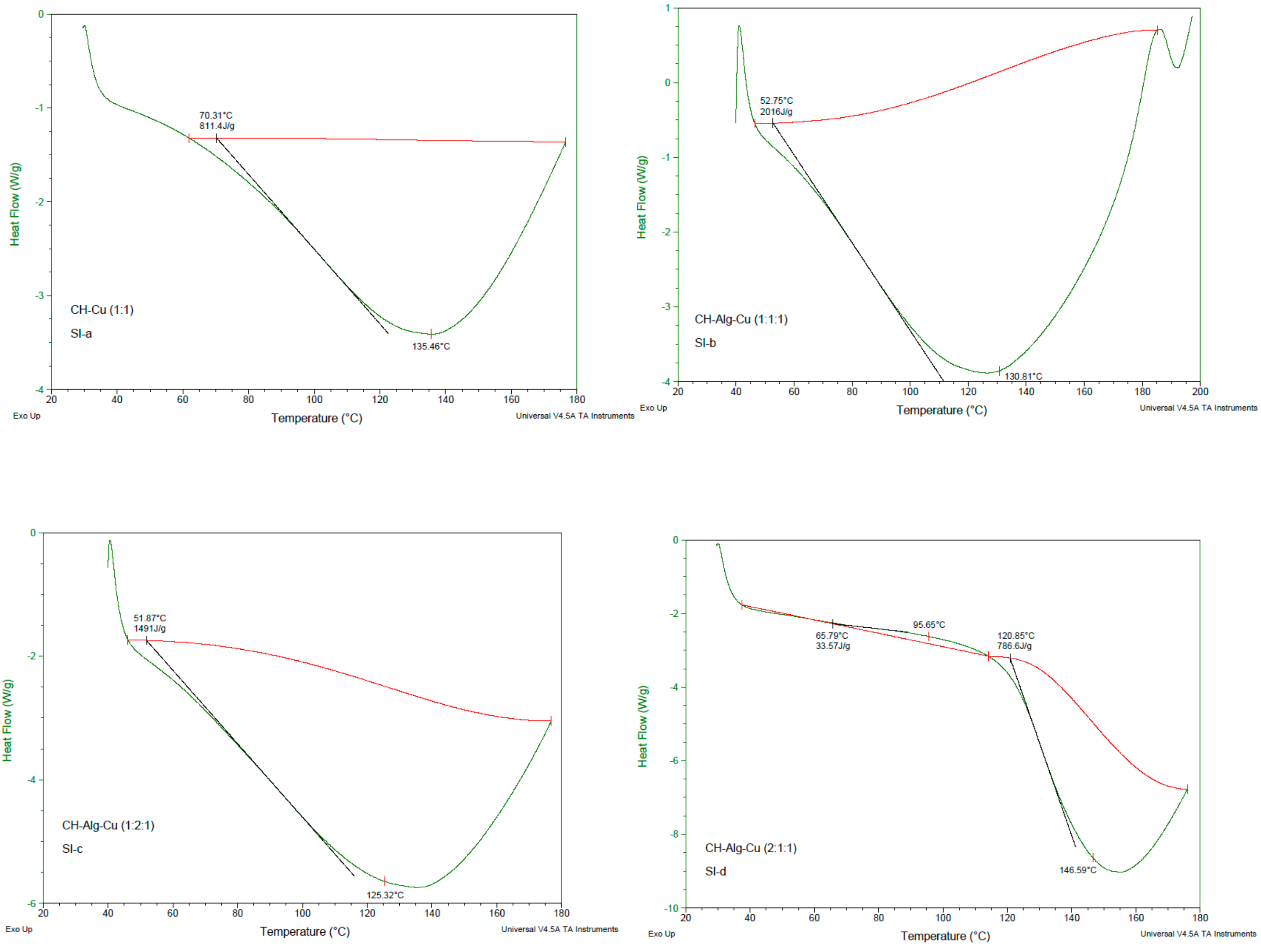


**Figure S1.** DSC thermograms of composite materials (a) CCu, (b) CACu1, (c) CACu2 and (d) CACu3.

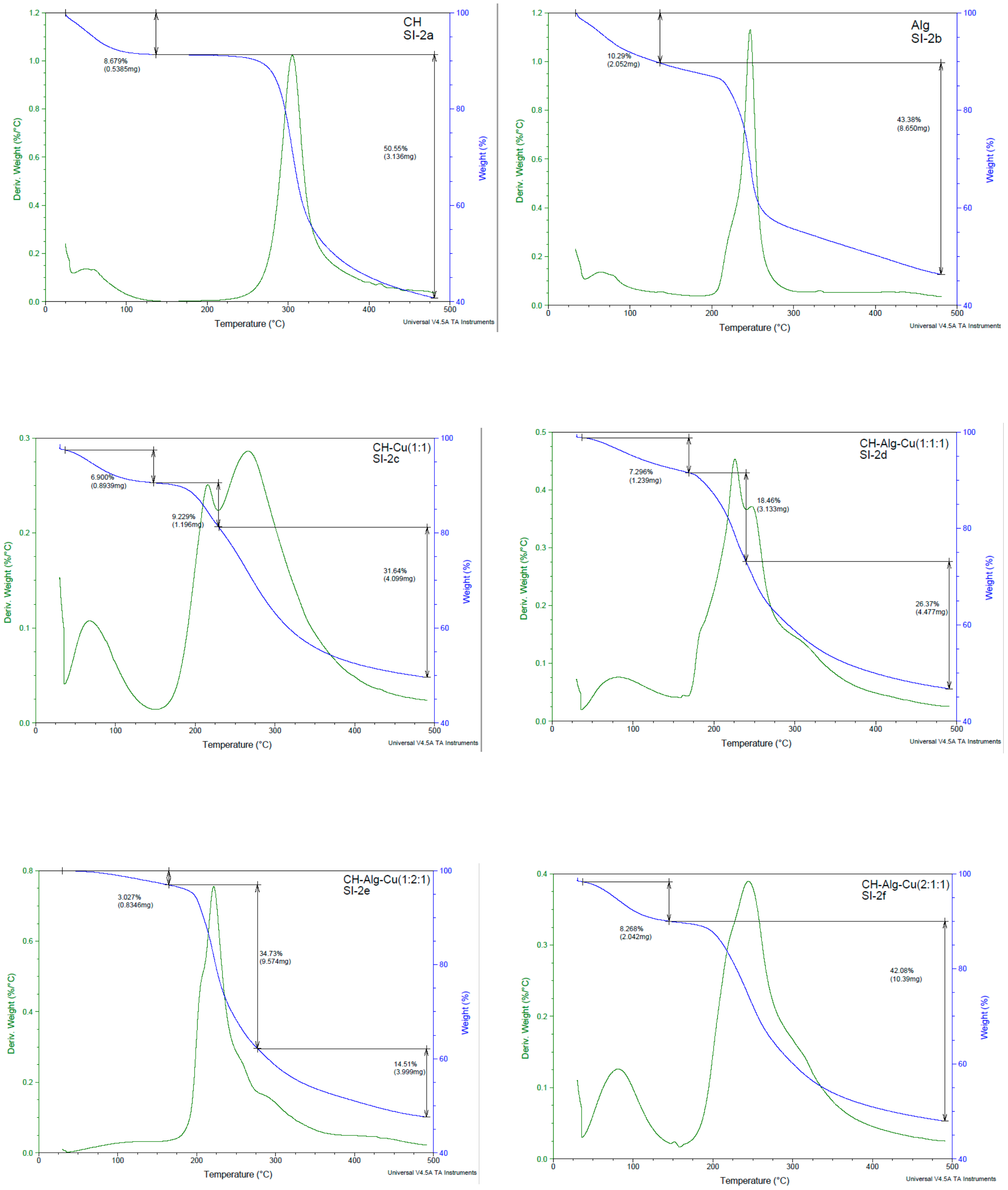


**Figure S2.** (**a–f**) Derivative plot from TGA for C, A, CCu, CACu1, CACu2, and CACu3.

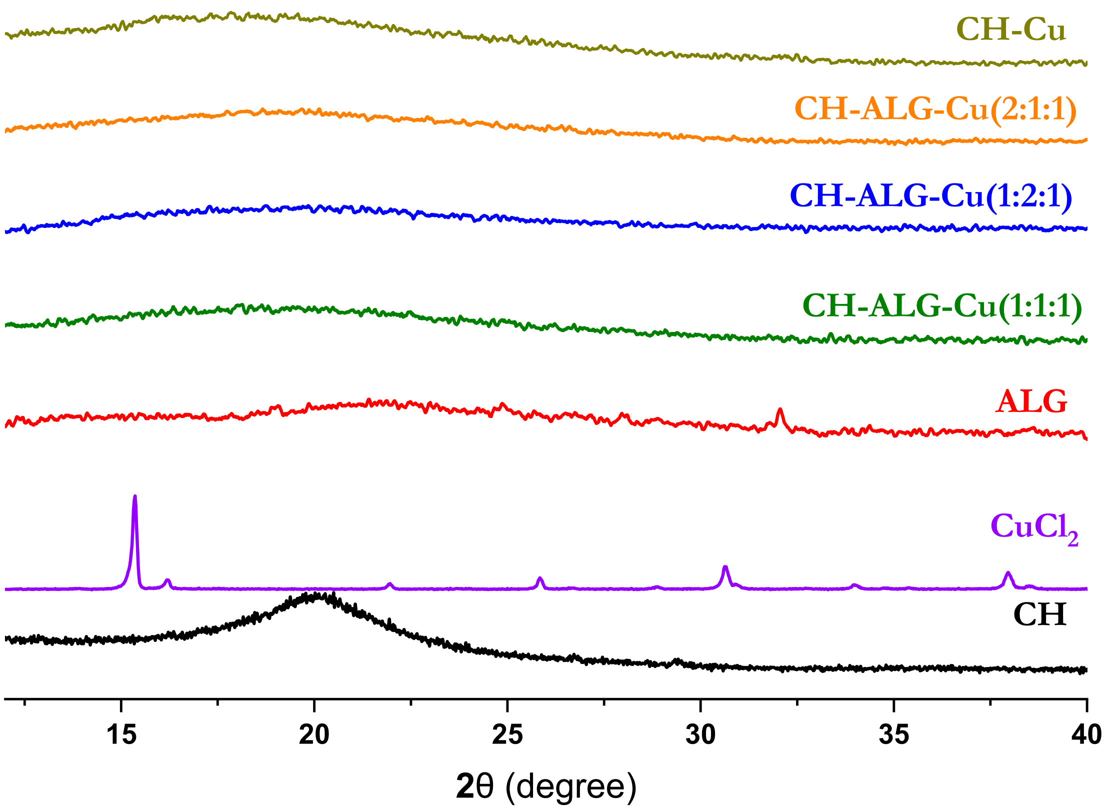


**Figure S3.** XRD patterns of C, A, CACu1, CACU2, CACu3 and CCu.

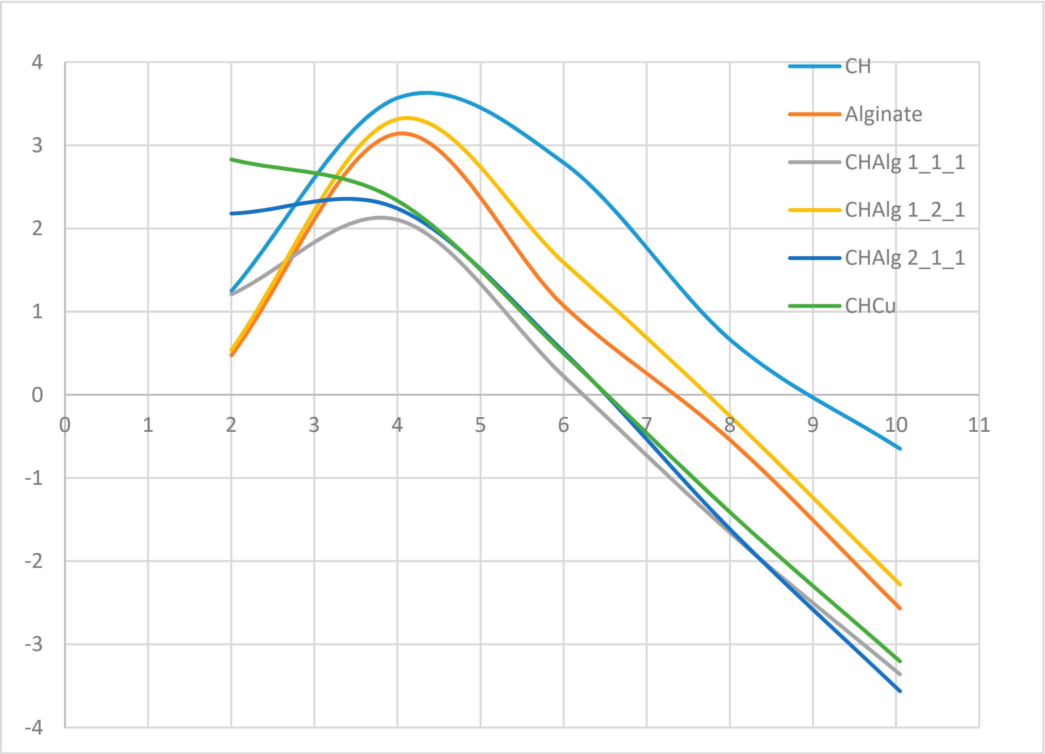


**Figure S4.** PZC graph for C, A, CCu and CACu1-3 materials.

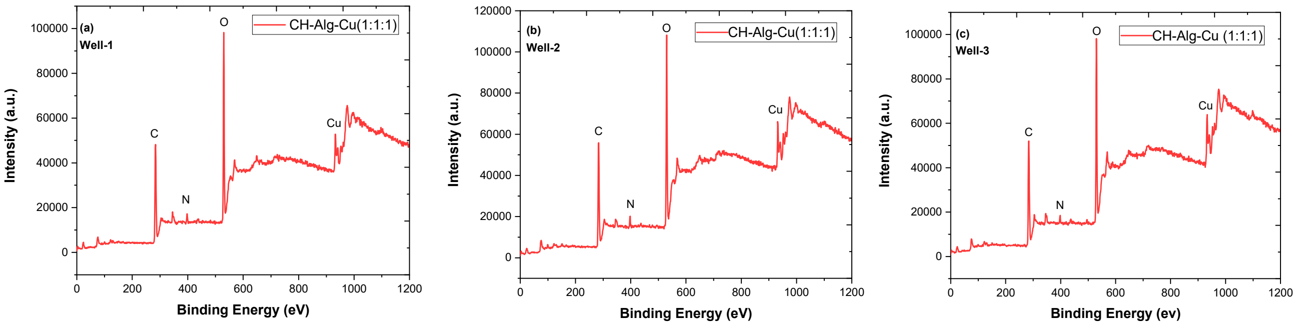


**Figure S5.** XPS wide scan spectra of CACu1 after the adsorption process.

**Table S5.** Measured (ICP-OES) copper concentration in well water samples and various composite materials (chitosan powder (C), chitosan pellet (CP), CAAl (chitosan-alginate-aluminum) and two CPCu (chitosan pellet; copper) as well as two CACu (chitosan, alginate, copper) materials) to assess potential health hazards from copper leaching of the polyelectrolyte composite materials and comparison to CBMS, where copper was replaced with aluminum to compare it with the copper containing materials concerning leaching.

| Source water | [Cu(II)] (cps) | RSD (%) | Reported (μg/L) |
|---|---|---|---|
| Well-1 | 44 | 24.2 | <8.29 |
| Well-2 | 32 | 9.9 | <8.29 |
| Well-3 | 46 | 12.4 | <8.29 |
| C | 12 | 31.0 | <8.29 |
| CP | 13 | 55.9 | <8.29 |
| CAA1 | 207 | 45.2 | <8.29 |
| CPCU7 | 3677 | 8.9 | <1000 |
| CPCU4 | 7228 | 0.9 | <1000 |
| CACu4 | 6565 | 2.5 | <1000 |
| CACu | 7268 | 4.7 | <1000 |
